# Functional Brain Hyperactivations Are Linked to an Electrophysiological Measure of Slow Interhemispheric Transfer Time after Pediatric Moderate/Severe Traumatic Brain Injury

**DOI:** 10.1089/neu.2019.6532

**Published:** 2019-12-20

**Authors:** Alexander Olsen, Talin Babikian, Emily L. Dennis, Monica U. Ellis-Blied, Christopher Giza, Sarah DeBoard Marion, Richard Mink, Jeffrey Johnson, Christopher J. Babbitt, Paul M. Thompson, Robert F. Asarnow

**Affiliations:** ^1^ Department of Psychiatry and Biobehavioral Sciences, Semel Institute for Neuroscience and Human Behavior, UCLA, Los Angeles, California.; ^2^ Department of Psychology, NTNU, Norwegian University of Science and Technology, Trondheim, Norway.; ^3^ Department of Physical Medicine and Rehabilitation, St. Olav's Hospital, Trondheim University Hospital, Trondheim, Norway.; ^4^ UCLA Steve Tisch BrainSPORT Program, Los Angeles, California.; ^5^ Psychiatry Neuroimaging Laboratory, Brigham & Women's Hospital, Boston, Massachusetts.; ^6^ Stanford Neurodevelopment, Affect, and Psychopathology Laboratory, Stanford, California.; ^7^ Imaging Genetics Center, Stevens Neuroimaging & Informatics Institute, Keck School of Medicine of USC, Marina del Rey, California.; ^8^ Fuller Theological Seminary School of Psychology, Pasadena, California.; ^9^ Loma Linda VA Healthcare System, Loma Linda, California.; ^10^ UCLA Mattel Children's Hospital, Los Angeles, California.; ^11^ Departments of Pediatrics and Neurosurgery, David Geffen School of Medicine at UCLA, UCLA, Los Angeles, California.; ^12^ Department of Psychology, Northwest Nazarene University, Nampa, Idaho.; ^13^ Elk's Rehabilitation Hospital, St. Luke's Health System, Boise, Idaho.; ^14^ Department of Pediatrics, Harbor-UCLA Medical Center and Los Angeles Biomedical Research Institute, Torrance, California.; ^15^ Department of Pediatrics LAC+USC Medical Center and Keck School of Medicine, Los Angeles, California.; ^16^ Miller Women's and Children's Hospital of Long Beach, Long Beach, California.; ^17^ Department of Psychology, UCLA, Los Angeles, California.; ^18^ Brain Research Institute, UCLA, Los Angeles, California.

**Keywords:** brain reserve, EEG, functional magnetic resonance imaging, interhemispheric transfer time, traumatic brain injury

## Abstract

Increased task-related blood oxygen level dependent (BOLD) activation is commonly observed in functional magnetic resonance imaging (fMRI) studies of moderate/severe traumatic brain injury (msTBI), but the functional relevance of these hyperactivations and how they are linked to more direct measures of neuronal function remain largely unknown. Here, we investigated how working memory load (WML)-dependent BOLD activation was related to an electrophysiological measure of interhemispheric transfer time (IHTT) in a sample of 18 msTBI patients and 26 demographically matched controls from the UCLA RAPBI (Recovery after Pediatric Brain Injury) study. In the context of highly similar fMRI task performance, a subgroup of TBI patients with slow IHTT had greater BOLD activation with higher WML than both healthy control children and a subgroup of msTBI patients with normal IHTT. Slower IHTT treated as a continuous variable was also associated with BOLD hyperactivation in the full TBI sample and in controls. Higher WML-dependent BOLD activation was related to better performance on a clinical cognitive performance index, an association that was more pronounced within the patient group with slow IHTT. Our previous work has shown that a subgroup of children with slow IHTT after pediatric msTBI has increased risk for poor white matter organization, long-term neurodegeneration, and poor cognitive outcome. BOLD hyperactivations after msTBI may reflect neuronal compensatory processes supporting higher-order capacity demanding cognitive functions in the context of inefficient neuronal transfer of information. The link between BOLD hyperactivations and slow IHTT adds to the multi-modal validation of this electrophysiological measure as a promising biomarker.

## Introduction

Increased task-related blood oxygen level dependent (BOLD) activation is the most common observation in functional magnetic resonance imaging (fMRI) studies of moderate-to-severe traumatic brain injury (msTBI). In general, BOLD activation typically increases with higher task load, as well as with other extrinsic factors (e.g., aging, sleep-wake disturbance, brain injury, and disease) that are associated with increased cerebral metabolic demand.^1,2^ In msTBI, BOLD hyperactivations are observed across different tasks and may represent injury-related compensatory mechanisms reflecting increased effort required to maintain task performance, inefficient neuronal processing, and/or merely changes in neurovascular coupling after injury.^1,3–7^ Task-elicited hyperactivations are the most consistent finding when fMRI task performance is maintained similarly between patients and controls, but some studies have also shown instances of hypoactivations in pediatric msTBI.^8,9^

Interpretation of fMRI findings in pediatric msTBI is complicated by the heterogeneous nature of msTBI pathology, small sample sizes, and the fact that few studies have linked BOLD alterations to clinical variables (e.g., Glasgow Coma Scale [GCS] score, time post-injury, and neuropsychological test measures), and none have investigated the relationship to more direct measures of neuronal functioning. Extant studies do not provide a clear understanding of the functional and clinical significance of BOLD alterations after msTBI in children and adolescents. To further increase our understanding of BOLD alterations after msTBI, the role of these alterations should not only be further validated by traditional neurocognitive and clinical measures, but also more direct measures of neuronal functioning.

Our previous work identified a subgroup of msTBI patients with slow interhemispheric transfer time (IHTT; a scalp visual event-related potential [ERP] measure of corpus callosum function).^10^ TBI patients with slow IHTT had poor white matter organization, long-term neurodegeneration, and poor cognitive outcome^10–13^ compared to msTBI patients with normal IHTT. Slow IHTT is therefore a promising electrophysiological biomarker in pediatric msTBI.^14^ Examining the relation between the electrophysiological IHTT measure and BOLD alterations will provide a significant step toward a more substantial understanding of the effect of msTBI on brain function, including determining whether alterations in the BOLD signal after msTBI reflect functional neuronal changes, and not just potential changes in neurovascular coupling.

Slow IHTT is a basic measure of neuronal efficiency that is likely to affect processing across several cognitive domains given that it is calculated based on the visual N1 component,^4^ which appears before ERPs related to domain-specific higher cognitive operations (e.g., P3).^5^ Slowed processing on a *behavioral* level has been suggested to partly explain BOLD hyperactivations after adult msTBI, but whether this actually represents recruitment of latent support mechanisms attributable to inefficient *neuronal* processing remains to be determined.^6,7^ Computationally demanding higher-order cognitive processes, such as working memory, are particularly dependent on coordinated processing in both hemispheres and, consequently, sensitive to altered IHTT.^8^ A clear advantage of using working memory tasks in fMRI research is that task load can be parametrically manipulated,^9^ while still preserving the opportunity to adjust for between-subject task performance by exclusively including correct trials when modeling the BOLD response.^10^ This provides a powerful approach to evaluating the consequences of higher cerebral demand^11,12^ as reflected by increased working memory load (WML)-dependent BOLD activation.

Taken together, both increased WML and slow behavioral response time after msTBI seem to represent increased demands on the brain, providing a need for recruiting extra neuronal resources to uphold task performance. After pediatric msTBI, it is likely that the consequences of slow IHTT and poor white matter organization become more evident with higher WML as the need to transfer and integrate information across the brain increases. In the context of accurate task performance, individuals with longer IHTT would therefore be expected to show BOLD hyperactivation as a function of higher WML.

Here we investigated spatial working memory task-related BOLD activations after pediatric msTBI in a subsample from the UCLA RAPBI (Recovery After Pediatric Brain Injury) study^10^ that had both fMRI and IHTT data available. Both higher WML and suboptimal neuronal transfer of information (operationalized as slow IHTT) as indices of increased cerebral demand^1,2^ were expected to be associated with increased BOLD activation. Specifically, we hypothesized that pediatric msTBI patients with slow IHTT would demonstrate a greater increase in BOLD activation as WML increased compared to msTBI individuals with normal IHTT and a healthy control group, possibly reflecting compensatory mechanisms. The external validity of BOLD alterations was further evaluated in the full msTBI sample by investigating associations with injury-related variables and a clinical measure of neurocognitive function.^20^

## Methods

### Participants

TBI patients were recruited from four different pediatric intensive care units located in level 1 and 2 trauma centers in Los Angeles County. Patients were included if they experienced a moderate-to-severe non-penetrating TBI, with intake or post-resuscitation GCS between 3 and 12 (or higher if there were confirmed abnormalities on clinical imaging), if they were between 8 and 18 years of age at the time of injury, had normal visual acuity or corrected vision with contact lenses or eyeglasses, and had sufficient English skills to understand instructions and participate in neurocognitive testing presuming English competence.

Participants were excluded if they had previous head injury, motor deficits precluding them from participating in the test protocols, or other significant pre-trauma history of neurological, developmental, or psychiatric disorders. Healthy control participants were matched on age, sex, and educational level to the TBI patients and were recruited from the community through flyers, magazines, and school postings. With the exception of criteria for TBI injury severity, healthy control participants met the same inclusion and exclusion criteria as TBI patients. Finally, participants were excluded from the MRI part of the study if they were not eligible for MRI (e.g., because of having metal implants that were not MRI compatible). The study was approved by the University of California, Los Angeles (UCLA) institutional review board and the institutional review boards of each site from which patients were recruited.

The study design has been described in greater detail elsewhere.^10^ Fifty patients were included in the overall study. However, because fMRI data collection was initiated late in the project, a total of 18 (4 female) patients had both task-fMRI and IHTT data collected at the same time point of sufficient quality to be included in this study. A group of 26 (11 female) demographically matched controls, with both task-fMRI and IHTT data, was also included. The TBI group was further divided into subgroups based on IHTT as previously described.^10^ Briefly, electroencephalography was recorded using a BIOSEMI system (BioSemi, Amsterdam, the Netherlands; sampling rate = 512 Hz, low-pass filter = 40Hz, high-pass filter = 0.16 Hz, bandwidth [3 dB] = 134 Hz) while participants performed a pattern matching task with bilateral field advantage. Stimuli for each trial were presented randomly and pairwise in two of four visual fields (upper and lower; right and left), creating four *bilateral* and two *unilateral* conditions.

Participants were asked to indicate whether the two stimuli (patterns) constituted a “match” or “non-match” by pressing a keyboard. The responding hand (right or left) was alternated in eight blocks of 97 trials each. ERPs time-locked to the stimulus presentation were calculated from parietal (P3/P4) and occipital (O1/O2) electrodes. The peak latency (in milliseconds) of the early visual N1 (typically observed ∼150–200 ms post-stimulus presentation) component was determined and averaged for the right and left hemisphere. An overall IHTT was calculated by averaging left to right and right to left differences (IHTTs) based on data from the *unilateral* conditions. Longer IHTT indicates slower transfer of visual information between the hemispheres, which is a basic and robust measure of neuronal efficiency, given that the N1 component reflects early visual registration occurring before any involvement of domain-specific higher cognitive operations, such as those reflected in *cognitive* ERPs, such as the P3.^5^

In the overall study, IHTT had a skewed distribution in the msTBI patients, with around half of the group having scores within 1.5 standard deviations (SDs) of the normal range, as calculated based on data from the healthy control group.^4^ The balance of the TBI group had slow IHTT outside the normal range (>1.5 SDs below the mean of the control group). The bimodal properties of the data therefore supported a meaningful cutoff for defining slow IHTT as being above 1.5 SDs of the mean of healthy controls.^4^ This cutoff has been successfully used in our earlier studies showing that TBI patients with slow IHTT had poor white matter organization, long-term neurodegeneration, and poor cognitive outcome.^4,13–15^ In the current study, subjects with an IHTT score of >1.5 SDs (≥18 ms) from the mean of the entire healthy control group that was included in the overall study^10^ were allocated to the “TBI slow” group (*n* = 7, 1 female), and the remaining patients were included in the “TBI normal” group (*n* = 11, 3 female). Demographic and clinical data for the groups are presented in Table 1.

**Table 1.** Demographics and Clinical Measures

Variable	F-statistics	Group	*n*	Mean (SD)	95% CI of means
Age	*F*_(2, 41)_ = 0.979, *p* = 0.384, η2 = 0.046	Controls	26	15.78 (3.12)	14.64, 16.92
		TBI normal	11	14.33 (2.67)	12.58, 16.09
		TBI slow	7	15.28 (2.09)	13.08, 17.48
IHTT average (msec)	*F*_(2, 41)_ = 36.70, *p* < 0.001, η2 = 0.642	Controls	26	7.58 (5.78)^*^	5.43, 9.73
		TBI normal	11	4.50 (3.75)^†^	1.20, 7.81
		TBI slow	7	25.50 (7.58)^^*^†^	21.36, 29.64
Cognitive Performance Index (standard score)	*F*_(2, 41)_ = 9.109, *p* = 0.001, η2 = 0.308	Controls	26	106.24 (9.77)^^*^†^	102.17, 110.30
		TBI normal	11	93.13 (11.54)^†^	86.88, 99.38
		TBI slow	7	92.32 (10.00)^*^	84.49, 100.16
GCS at admission	—	TBI normal	11	9.00 (3.35)	6.89, 11.11
		TBI slow	7	9.00 (3.21)	6.36, 11.64
Time post-injury (weeks)	—	TBI normal	11	38.08 (31.64)	19.51, 58.18
		TBI slow	7	38.85 (29.38)	12.85, 61.32

Footnote symbols (^*^ or †) = pair-wise statistically significant difference, *p* < 0.05.

IHTT, interhemispheric transfer time; TBI, traumatic brain injury; GCS, Glasgow Coma Scale; SD, standard deviation; CI, confidence interval; ANOVA, analysis of variance; η2, partial eta squared.

### Clinical measure of neurocognitive function

A previously validated cognitive performance index (CPI) was included as a clinical measure of neurocognitive function.^20^ This index combined standardized scores from tests measuring verbal memory, psychomotor skills, working memory, and inhibition/set-switching into a single psychometrically sound summary score representing overall cognitive efficiency.^20^

### Analyses of demographic and clinical measures

Means, SDs, and 95% confidence intervals (CIs) of the means were calculated (Table 1). For measures where all groups had data, separate analyses of variance (ANOVAs) with group (TBI slow, TBI normal, and controls) as a between-subject factor and each dependent variable as a within-group factor were performed. Statistically significant main effects were followed up by pair-wise between-group contrasts. For TBI-related measures, pair-wise comparisons using an independent *t*-test were applied. The acceptance level for statistically significant results was set to *p* < 0.05, and partial eta squared (ηρ^2^) was calculated as a measure of effect size.

IBM SPSS Statistics Version 24 was used for statistical analysis of demographic, clinical, and behavioral data.

### Spatial working memory task

BOLD fMRI was acquired during performance of a parametric spatial working memory task. Before each trial, four squares appeared on the screen, indicating the spatial locations where stimuli could appear. After each trial, these squares were replaced with triangles, indicating that the participant should start responding. For each trial, one to five pictures (items) of fruits and vegetables were sequentially presented for 800 ms within the four possible different locations on the screen. Interstimulus time was 200 ms, and intertrial time was 1500 ms (from the last response to the beginning of a new sequence). Subjects were instructed to attend to the four positions on the screen and reproduce the order of the presentation (not what pictures were presented) by using a response box with four buttons (Current Designs, Winona, MN) corresponding to the respective spatial locations. Because several similar objects were presented (e.g., 25 different apples), respondents were not able to associate a unique word with each stimulus, thereby minimizing verbal mediation as a working memory strategy.

Participants were asked to respond as quickly and accurately as possible. All participants completed a practice session before the actual fMRI using a mock setup before their scan to ensure understanding of task instructions.

Task demands were manipulated in a parametric manner giving four trial types with different WML: baseline (one item), WML 3 (three items), WML 4 (four items), and WML 5 (five items). The task consisted of a total of 128 trials divided into 32 trials of each trial type, divided into four equally long time epochs that were balanced with regard to WML and intratrial sequence order to avoid potential order effects. Stimuli were presented through a head-coil mounted mirror system and an in-house custom-built MRI compatible video projector (Staglin Center, UCLA). The task design was implemented using the PsychToolbox in Matlab (The MathWorks, Inc., Natick, MA). A log file containing behavioral data was stored, and response time and the number of correct responses were computed separately for each WML as measures of accuracy and response speed.

### Analysis of functional magnetic resonance imaging behavioral data

Means and SDs for each behavioral measure were calculated separately for the respective groups (Table 2). To investigate within- and between-group effects of WML, separate repeated-measures ANOVAs with group (TBI slow, TBI normal, and controls) as the between-subject factor and WML (baseline, WML 3, WML 4, and WML 5) as the within-group factor were performed. Mauchley's test was used to investigate the assumption of sphericity of the data, and a Greenhouse-Geisser correction was used if this assumption was violated. Statistically significant main effects were followed up by pair-wise between-group contrasts. Subsequent polynomial trend analyses were used to further evaluate the expected linear WML effects. The acceptance level for statistically significant results was set to *p* < 0.05. Partial eta squared (ηρ^2^) was calculated as a measure of effect size.

**Table 2.** Working Memory fMRI Task Performance

Variable	Group	*n*	Baseline	WML 3	WML 4	WML 5
			mean (SD)	mean (SD)	mean (SD)	mean (SD)
Accuracy (correct responses)	Controls	26	31.19 (1.88)	26.92 (4.87)	24.65 (5.75)	22.00 (7.38)
	TBI normal	11	30.81 (1.47)	24.64 (5.97)	22.73 (6.08)	17.91 (7.11)
	TBI slow	7	31.14 (1.21)	26.57 (4.69)	25.29 (3.15)	21.71 (4.79)
Response speed (seconds)	Controls	26	0.814 (.245)	1.637 (0.378)^*^	2.116 (0.533)^*^	2.784 (1.109)
	TBI normal	11	1.113 (.594)	2.156 (0.523)^*^	2.766 (0.730)^*^	3.256 (0.772)
	TBI slow	7	0.764 (.065)	1.731 (0.405)	2.169 (0.406)	2.846 (0.649)

^*^ Pair-wise statistically significant difference, *p* < 0.05.

fMRI, functional magnetic resonance imaging; WML, working memory load; SD, standard deviation; TBI, traumatic brain injury; IHTT, interhemispheric transfer time.

### Scan acquisition

All scans were performed on a Siemens Trio with a 12-channel Head Matrix Coil (Siemens AG, Erlangen, Germany). Foam pads around the subjects' heads were used to reduce head motion. During the parametric spatial working memory task, T2*-weighted BOLD fMRI was acquired utilizing an echo-planar imaging pulse sequence (repetition time = 2400 ms, echo time = 35 ms, field of view = 244 mm, matrix = 80 × 80, slice thickness = 3 mm, number of slices = 40, giving an in-plane resolution of 3 × 3 mm). A T1-weighted three-dimensional magnetization-prepared rapid gradient-echo (MPRAGE) volume was acquired for anatomical reference.

### Analysis of magnetic resonance imaging data

All analyses were performed using the FMRIB's Software Library (FSL) toolbox (version 5.0.7; FMRIB Centre, Oxford, UK). Non-brain structures were removed with BET.^21^ The fMRI data were motion corrected with MCFLIRT,^22^ smoothed (Gaussian kernel full width at half maximum, 6 mm), grand mean normalized, high-pass temporal filtered (50 sec), and linearly registered to native high-resolution space (T1 MPRAGE), using a 7 degrees of freedom spatial transformation.^22, 23^ A transformation matrix was created by registration of the high-resolution T1 MPRAGE to a 2-mm Montreal Neurological Institute (MNI) standard template using 12 degrees of freedom, and fMRI data were subsequently transformed into standard MNI space by applying the same transformation matrix.

BOLD activation was modeled using a general linear model, and the hemodynamic response function was convolved with a standard Gamma variate. A main contrast was computed to model the linear increase of BOLD activation as a function of WML (baseline, WML 3, WML 4, and WML 5). It is important for the validity of fMRI studies comparing patient and healthy control groups that task performance in the included contrasts is highly similar.^19^ Therefore, only trials with correct performance were included. First, all fMRI contrasts were computed for each participant using a fixed-effects model. Then, mixed-effects models with automatic outlier deweighting were used to create statistical parametric maps (SPMs) and to investigate group differences and within-group associations. The main analysis focused on investigating differences between the predefined IHTT groups: TBI slow, TBI normal, and healthy controls.

To substantiate our findings, we evaluated the clinical and functional relevance of the BOLD activations by performing within-group analyses for the full TBI sample, evaluating associations with age, IHTT (as a continuous variable), the CPI, injury severity (GCS score at admission), and time post-injury (number of weeks). In the control group, we evaluated associations with age, IHTT, and the clinical neurocognitive performance index.

All SPMs were corrected for multiple comparisons by applying a cluster threshold of *Z* > 2.3 and a cluster significance threshold of *p* < 0.05. Peak *Z*-values with up to five local maxima and the size of clusters (number of voxels) in standard 2 × 2 × 2 mm MNI space were extracted. For anatomical denotation, visual inspection and the Harvard Oxford cortical and subcortical structural brain atlases as incorporated in FSL were applied.

## Results

### Demographic and clinical data

Results are presented in Table 1. There were no statistically significant differences in age between the TBI slow, TBI normal, and control groups. As expected, the TBI slow group had slower IHTT than both the TBI normal group and controls, but there was no statistically significant difference in IHTT between the TBI normal group and controls. Both TBI groups performed worse than controls on the clinical neurocognitive performance index, but did not differ significantly from each other. There was also no statistically significant difference between the TBI groups for injury severity, nor time post-injury.

### Behavioral functional magnetic resonance imaging data

**FIG. 1.**
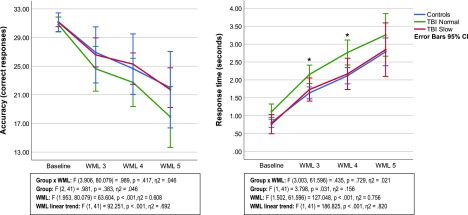
Working memory fMRI task performance. *Pair-wise statistically significant difference between the TBI normal group and controls, *p* < 0.05. CI, confidence interval; fMRI, functional magnetic resonance imaging; TBI, traumatic brain injury; WML, working memory load.

Means, SDs, and pair-wise comparisons for accuracy and response speed are presented in Table 2, and results are also plotted in Figure 1. There were no statistically significant WML × group interactions for any of the behavioral measures. There were, however, statistically significant main effects of WML, revealing a linear decrease in performance as measured by response speed and accuracy with higher WML. The only statistically significant effect of group was found for response speed at WML 3 and 4, where the TBI normal group had slower response times than controls.

### Blood oxygen level dependent functional magnetic resonance imaging data

**FIG. 2.**
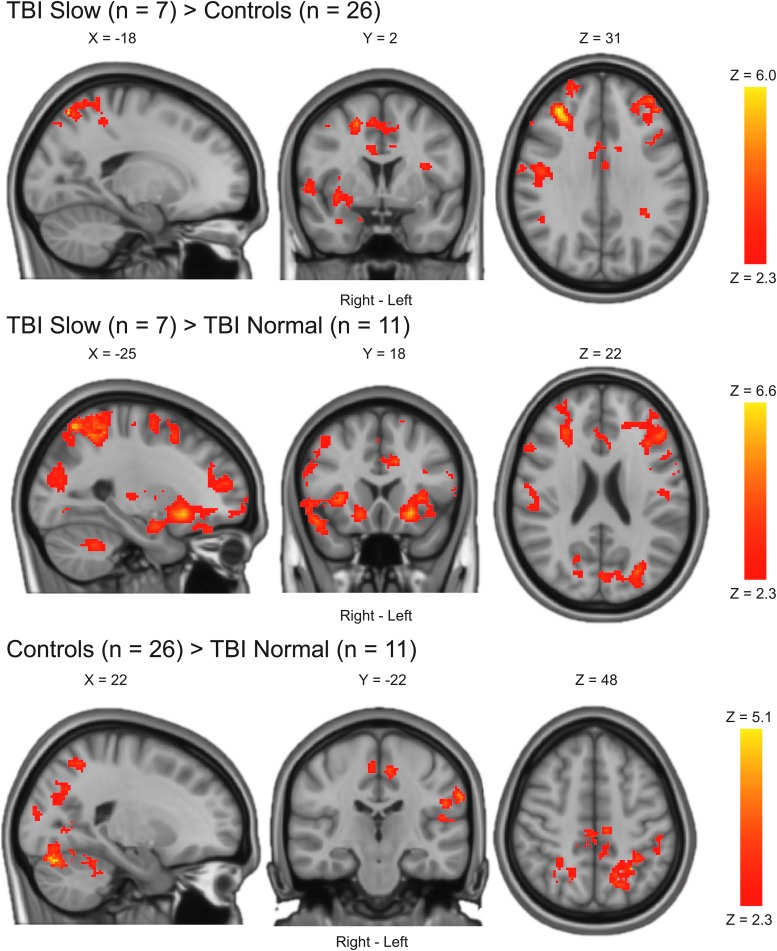
Between-group differences in BOLD activation. Statistical parametric maps were achieved using a mixed-effects model corrected for multiple comparisons using a cluster threshold of Z > 2.3 and a corrected cluster significance threshold of *p* < 0.05. Only statistically significant results are shown. There were no regions where the TBI normal group or controls had statistically significant higher BOLD activation than the TBI slow group. There were no regions where the TBI normal group had statistically significant higher BOLD activation than controls. BOLD, blood oxygen level dependent; TBI, traumatic brain injury.

The TBI slow group exhibited BOLD hyperactivation as compared to both the TBI normal group and controls in widespread brain areas encompassing frontal, parietal, and occipital regions (Table 3; Fig. 2). There were *no regions* where the TBI normal group or controls had statistically significantly higher BOLD activation than the TBI slow group. The TBI normal group exhibited BOLD *hypoactivation* compared to controls, predominantly in posterior brain regions (Table 3; Fig. 2). There were *no regions* where the TBI normal group had statistically significantly higher BOLD activation than controls.

**Table 3.** Between-Group Differences in BOLD Activation

Anatomical region	R/L	Size in number of voxels	Z	Coordinates for peak activation (MNI)		
				X	Y	Z
TBI slow > control						
Frontal pole	R	6603	6.01	34	38	30
Frontal pole	R	lm	5.75	32	38	34
Supramarginal gryus, anterior division	R	lm	5.48	58	–24	50
Inferior frontal gyrus, pars triangularis	R	lm	5.46	58	26	–4
Frontal pole	R	lm	5.40	36	38	34
Middle frontal gyrus	R	lm	5.39	32	34	36
Lateral occipital cortex, superior division	L	1644	5.67	–18	–70	58
Lateral occipital cortex, superior division	L	lm	5.11	–16	–78	50
Superior parietal lobule	L	lm	4.76	–22	–46	58
Superior parietal lobule	L	lm	4.65	–22	–48	62
Superior parietal lobule	L	lm	4.55	–28	–46	58
Lateral occipital cortex, superior division	L	lm	4.50	–10	–66	60
Frontal pole	L	978	4.66	–36	46	32
Insular cortex	L	lm	4.43	–30	18	6
Middle frontal gyrus	L	lm	4.29	–42	38	30
Middle frontal gyrus	L	lm	4.09	–32	34	36
Middle frontal gyrus	L	lm	4.05	–30	24	36
Frontal pole	L	lm	3.83	–40	44	20
Superior frontal gyrus	R	930	4.37	20	2	52
Cingulate gyrus, anterior division	L	lm	3.89	–4	8	40
Supplementary motor cortex	R	lm	3.83	6	−8	54
Supplementary motor cortex	L	lm	3.69	–10	0	50
Supplementary motor cortex	R/L	lm	3.68	0	4	52
Supplementary motor cortex	R	lm	3.62	6	–2	54
TBI slow > TBI normal						
Lateral occipital cortex, superior division	L	17,424	6.50	–22	–68	58
Lateral occipital cortex, superior division	L	lm	5.81	–18	–70	60
Lateral occipital cortex, superior division	L	lm	5.49	–12	–68	60
Precuneus cortex	L	lm	5.35	–6	–68	54
Postcentral gyrus	R	lm	5.30	12	–46	72
Superior parietal lobule	L	lm	5.27	–22	–46	58
Frontal orbital cortex	L	5891	5.63	–24	18	–10
Precentral gyrus	L	lm	4.93	–56	4	4
Middle frontal gyrus	L	lm	4.59	–36	24	26
Insular cortex	L	lm	4.49	–30	16	2
Precentral gyrus	L	lm	4.47	–54	4	34
Insular cortex	L	lm	4.46	–32	16	6
Lateral occipital cortex, superior division	L	3513	4.58	–28	–76	22
Intracalcarine cortex	L	lm	4.42	–6	–86	6
Lateral occipital cortex, superior division	L	lm	4.12	–32	–78	18
Occipital pole	L	lm	3.99	–8	–96	4
Intracalcarine cortex	R	lm	3.97	14	–80	6
Cerebellum (left VI)	L	lm	3.93	–24	–52	–34
Controls > TBI normal						
Occipital fusiform gyrus	R	4709	5.09	22	–76	–22
Cuneal cortex	R	lm	4.72	10	–80	32
Cuneal cortex	R	lm	4.68	10	–76	32
Occipital fusiform gyrus	R	lm	4.47	26	–76	–18
Lateral occipital cortex, superior division	R	lm	4.45	20	–62	54
Lateral occipital cortex, superior division	L	lm	2.26	–30	–66	20
Postcentral gyrus	L	1285	4.13	–62	–22	32
Parietal operculum cortex	L	lm	3.99	–50	–26	14
Planum temporale	L	lm	3.95	–44	–34	14
Supramarginal gyrus, anterior division	L	lm	3.88	–46	–36	42
Superior parietal lobule	L	lm	3.83	–36	–42	64
Supramarginal gyrus, anterior division	L	lm	3.81	–50	–38	42
Superior parietal lobule	L	949	4.52	–28	–54	48
Lateral occipital cortex, superior division	L	lm	4.00	–28	–58	60
Superior parietal lobule	L	lm	3.90	–20	–52	50
Superior parietal lobule	L	lm	3.88	–30	–46	44
Superior parietal lobule	L	lm	3.84	–24	–56	50
Superior parietal lobule	L	lm	3.81	–30	–54	56
Controls > TBI slow	—	—	—	—	—	—
TBI normal > controls	—	—	—	—	—	—
TBI normal > TBI slow	—	—	—	—	—	—

Results were achieved using a mixed-effects model corrected for multiple comparisons using a cluster threshold of Z > 2.3 and a corrected cluster significance threshold of *p* < 0.05. Main peaks and up to five local maxima (lm) within each cluster are reported. Naming of anatomical regions was based on the Harvard Oxford cortical and subcortical structural atlases as implemented in the FSL software. Note that some clusters are relatively large and therefore span over several brain regions (see Fig. 1 for more details).

BOLD, blood oxygen level dependent; TBI traumatic brain injury; MNI, Montreal Neurological Institute.

**FIG. 3.**
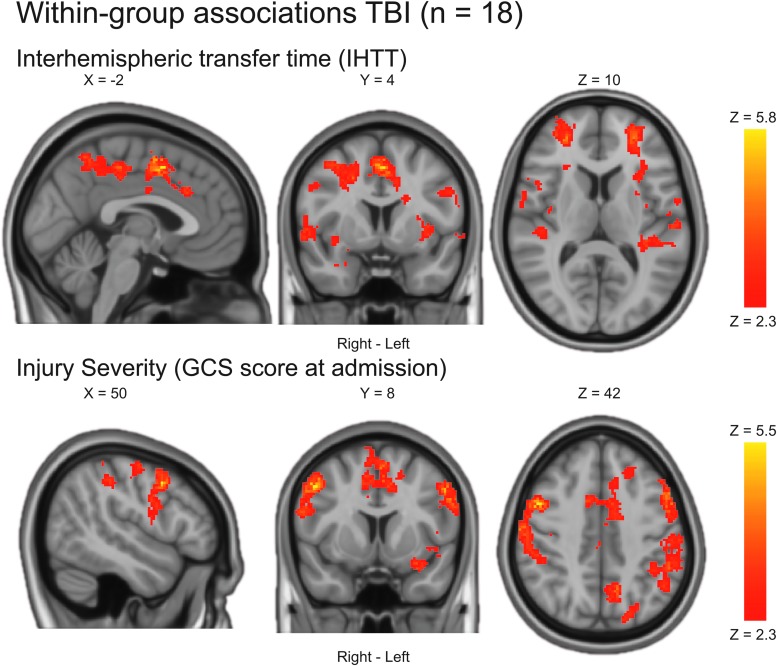
Within-group associations in the full TBI sample. Statistical parametric maps were achieved using a mixed-effects model corrected for multiple comparisons using a cluster threshold of Z > 2.3 and a corrected cluster significance threshold of *p* < 0.05. Only statistically significant results are shown. There was no statistically significant association between BOLD activation and age, time post-injury, nor the clinical neurocognitive performance index in the TBI group. BOLD, blood oxygen level dependent; GCS, Glasgow Coma Scale; IHTT, interhemispheric transfer time; TBI, traumatic brain injury.

**FIG. 4.**
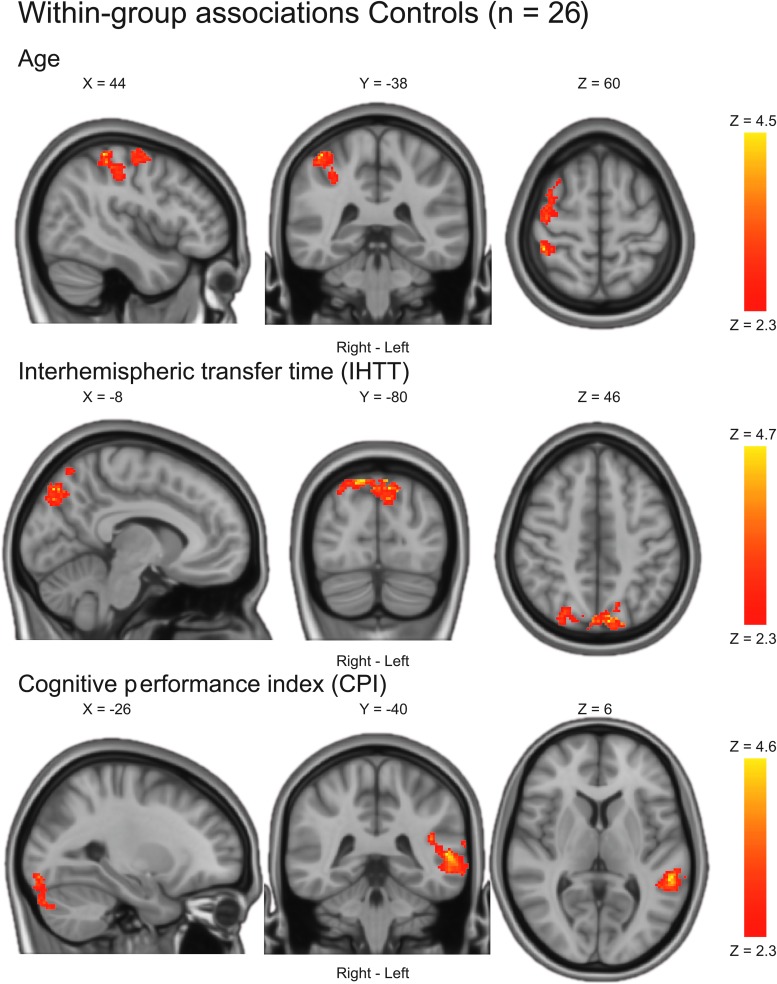
Within-group associations in controls. Statistical parametric maps were achieved using a mixed-effects model corrected for multiple comparisons using a cluster threshold of Z > 2.3 and a corrected cluster significance threshold of *p* < 0.05. Only statistically significant results are shown.

In the full TBI sample, slower IHTT (treated as a continuous variable) was associated with increased BOLD activation in widespread brain regions (Table 4; Fig. 3). Less severe injury (as measured by GCS at admission) was also associated with increased BOLD activation (Table 4; Fig. 3). The associations between BOLD activation and age, time post-injury, or the clinical neurocognitive performance index in the TBI group were not statistically significant. In controls, older age was associated with increased BOLD activation in predominantly right posterior brain regions (Table 4; Fig. 4). Also, in controls, better neurocognitive function as measured by the CPI was associated with increased BOLD activation in left temporo-occipital regions (Table 4; Fig. 4). Slower IHTT in controls was associated with increased BOLD activation bilaterally in occipitoparietal regions (Table 4; Fig. 4).

**Table 4.** Within-Group Associations

Anatomical region	R/L	Size in number of voxels	Z	Coordinates for peak activation (MNI)		
				X	Y	Z
TBI (*n* = 18)						
Age	—	—	—	—	—	—
Cognitive performance index (CPI)	—	—	—	—	—	—
Interhemispheric transfer time (IHTT)						
Supplementary motor cortex	L	13,621	5.74	–2	4	50
Superior parietal lobule	L		5.05	–26	–52	52
Superior parietal lobule	R		4.95	40	–44	54
Supramarginal gyrus, posterior division	R		4.92	46	–44	58
Supplementary motor cortex	L		4.87	–2	0	56
Lateral occipital cortex, superior division	L		4.84	–16	–70	58
Frontal pole	L	1673	4.42	–26	48	10
Middle frontal gyrus	L		4.11	–36	30	30
Middle frontal gyrus	L		4.11	–34	24	26
Middle frontal gyrus	L		3.67	–40	34	28
Middle frontal gyrus	L		3.66	–30	34	38
Frontal orbital cortex	L		3.63	–24	18	–10
Injury severity (GCS at admission)						
Precentral gyrus	L	12,077	5.42	–54	–4	46
Middle frontal gyrus	L		5.29	–50	28	38
Frontal pole	R		5.07	28	36	28
Frontal pole	L		5.04	–32	52	18
Precentral gyrus	L		5.01	–52	6	40
Middle frontal gyrus	R	1933	5.37	50	8	42
Precentral gyrus	R		5.26	58	6	38
Postcentral gyrus	R		4.58	64	–14	40
Middle frontal gyrus	R		4.40	46	8	48
Supramarginal gyrus, posterior division	R		4.06	52	–36	56
Postcentral gyrus	R		3.93	60	–16	44
Time post-injury	—	—	—	—	—	—
Controls (*n* = 26)						
Age						
Superior parietal lobule	R	1288	4.49	44	–38	60
Postcentral gyrus	R		3.94	44	–36	54
Postcentral gyrus	R		3.92	52	–28	52
Precentral gyrus	R		3.73	32	–18	68
Superior parietal lobule	R		3.70	40	–40	56
Postcentral gyrus	R		3.62	42	–32	52
IHTT						
Lateral occipital cortex, superior division	L		4.66	–8	–80	46
Precuneous cortex	R		4.53	6	–74	52
Lateral occipital cortex, superior division	R		4.48	14	–80	52
Lateral occipital cortex, superior division	R		4.39	18	–80	50
Precuneous cortex	L		4.25	–8	–76	46
Precuneous cortex	L		4.16	–6	–80	52
CPI						
Occipital fusiform gyrus	L	869	3.68	–26	–90	–12
Occipital pole	L		3.58	–22	–94	–14
Occipital fusiform gyrus	L		3.53	–24	–90	–18
Inferior temporal gyrus, temporooccipital part	L		3.48	–52	–54	–28
Temporal occipital fusiform cortex	L		3.48	–36	–56	–22
Cerebellum, left crus I	L		3.48	–28	–84	–32
Superior temporal gyrus, posterior division	L	728	4.52	–54	–40	6
Supramarginal gyrus, posterior division	L		3.88	–48	–46	22
Planum temporale	L		3.69	–42	–40	18
Middle temporal gyrus, posterior division	L		3.56	–60	–38	–6
Middle temporal gyrus, posterior division	L		3.55	–56	–40	–6
Parietal operculum cortex	L		3.54	–44	–44	24

Results were achieved using a mixed-effects model corrected for multiple comparisons using a cluster threshold of Z > 2.3 and a corrected cluster significance threshold of *p* < 0.05. Main peaks and up to five local maxima (lm) within each cluster are reported. Naming of anatomical regions was based on the Harvard Oxford cortical and subcortical structural atlases as implemented in the FSL software. Note that some clusters are relatively large and therefore span over several brain regions (see Figs. 3 and 4 for more details).

TBI traumatic brain injury; GCS, Glasgow Coma Scale; MNI, Montreal Neurological Institute.

## Discussion

This study demonstrated that BOLD hyperactivation in children with msTBI is associated with an electrophysiological ERP measure of IHTT. A subgroup of msTBI children with slow IHTT had greater BOLD activation with higher WML than both healthy control children and a subgroup of msTBI children with normal IHTT. The same effect was also demonstrated in the full TBI sample, where slower IHTT modeled as a continuous variable was associated with widespread BOLD hyperactivations. BOLD hyperactivations are commonly observed after msTBI, and here we provide the first compelling evidence on how such activations are linked to a more direct measure of neuronal function.

Increased BOLD activations are hypothesized to represent increased metabolic demands in the brain. These can arise from increased task load (e.g., WML), but also from suboptimal neuronal transfer of information,^1,2^ which in our study was operationalized as reduced IHTT. Our main contrast of interest was the linear increase in activation with higher WML in a parametric fMRI design. Behavioral results confirmed the expected WML-dependent linear decrease in accuracy and increase in response latency. In the fMRI analyses, all groups also demonstrated the expected effect, with more pronounced BOLD activations with greater WML.

BOLD hyperactivation in TBI individuals with slower IHTT may indicate that they had to recruit more neuronal resources to maintain task performance comparable to that of controls, as the working memory load increased. This likely reflects the presence of compensatory mechanisms; processes that in this study may have mitigated the effect of brain injury on cognitive performance during fMRI. A link between slower IHTT and increased BOLD activation was also confirmed within the healthy control group. Importantly, there were no group × WML interaction effects for online fMRI task performance, given that this could potentially compromise the interpretation of BOLD group comparisons.^19^ To further minimize any effects of more subtle performance differences, we only included correct trials in our analyses. Given that there were no demographic or clinical differences between the slow and normal IHTT groups, the BOLD hyperactivations in the msTBI children with slow IHTT is not likely to be explained by poor effort, demographics, or injury-related differences.

Neurovascular coupling can be affected after brain injury, potentially even in the chronic phase,^4,7^ which has provided some uncertainty about the interpretation of the functional role of BOLD alterations after msTBI. Here, we took an alternative approach to determining the role of BOLD alterations in msTBI by evaluating their link to IHTT—a basic electrophysiological measure of neuronal function. No previous studies have investigated associations between BOLD fMRI and electrophysiological data in msTBI. In a study combining data from adults with mild TBI and matched healthy controls, decreased amplitude in the event-related N350 component derived from a working memory task was associated with smaller BOLD signal changes in a corresponding fMRI task.^1^ However, this association could not be reproduced separately in the mild TBI group, leaving it an open question whether this association was linked to the brain injury or not. Longer IHTT is associated with slower transfer of visual information across posterior visual brain regions, particularly relying on pathways in the posterior corpus callosum.^25^ Higher-order cognitive processes such as working memory—and in particular those that are computationally demanding (e.g., higher WML)—rely on coordinated processing nodes in both hemispheres.^18^ A possible explanation of our findings is that impaired IHTT after msTBI may reflect disrupted long-range interhemispheric collaboration, which, in turn, causes increased reliance on local or core networks. Support for this has been found in studies observing decreased global connectivity in the context of increased local connectivity after msTBI.^26,27^ Moreover, in adult TBI, hyperconnectivity seems to occur in what is typically observed to be the most highly connected core regions in the brain, also referred to as the “rich club.”^28^ The rich club is a high-cost, high-capacity feature of brain connectivity central for global brain communication that encompass brain regions such as the anterior/posterior cingulate cortex, superior frontal cortex, the insula, and precuneus,^29^ some of which were implicated in the current study.

Interestingly, our preliminary analyses of structural connectivity in an overlapping sample show that slow IHTT is, in fact, linked to rich club hyperconnectivity at the expense of reduced peripheral integrity.^30^ An alternative, but non-mutually exclusive, mechanism is that the BOLD alterations observed in the current study are caused by a more global effect of white matter injury, beyond the corpus callosum. Reduced corpus callosum integrity after msTBI is typically indicative of more widespread white matter disorganization attributable to traumatic axonal injury caused by acceleration/deceleration and rotational forces.^31^

Despite examples of studies in pediatric msTBI examining relevant cognitive constructs (e.g., working memory) through the design of the fMRI task, few have investigated links to external clinical measures of neurocognitive function. Previous studies including individuals with pediatric msTBI have indicated both positive and negative relationships between BOLD activations and external measures of neurocognitive function.^2,3^ However, these results cannot be directly attributed to msTBI, given that orthopedic controls^2^ or patients with other types of acquired brain injuries^3^ were also included in the analyses. Cognitive dysfunction is common after pediatric msTBI,^20,33^ and was also confirmed in our subsample, because both TBI groups performed worse than controls on a clinical CPI. In controls, better performance on the CPI was associated with more pronounced BOLD activation in temporo-occipital regions. However, we failed to confirm a direct link between BOLD activation and the clinical neurocognitive performance index for the TBI group as a whole.

Brain reorganization and compensatory mechanisms are expected to develop over time as spontaneous recovery and experience-dependent new learning occurs.^1,34^ Relatively large variability in time post-injury for the subset of patients included in the fMRI analyses could therefore potentially have affected the BOLD-function results. This is unlikely, however, given that we found no significant association between time post-injury and BOLD activation. Differences in task demands between the fMRI task and the CPI (which is a broader measure of cognitive function) may also have obscured the BOLD-function relationship, given that compensatory mechanisms after msTBI may be more relevant for some tasks than others. Performance was highly similar across all groups in the spatial working memory task, and we only included correct responses in the fMRI analyses. Although important for the interpretation of BOLD activations,^19^ similar task performance may also indicate that the task difficulty was lower in the fMRI task than for the tasks combined in the CPI.

**FIG. 5.**
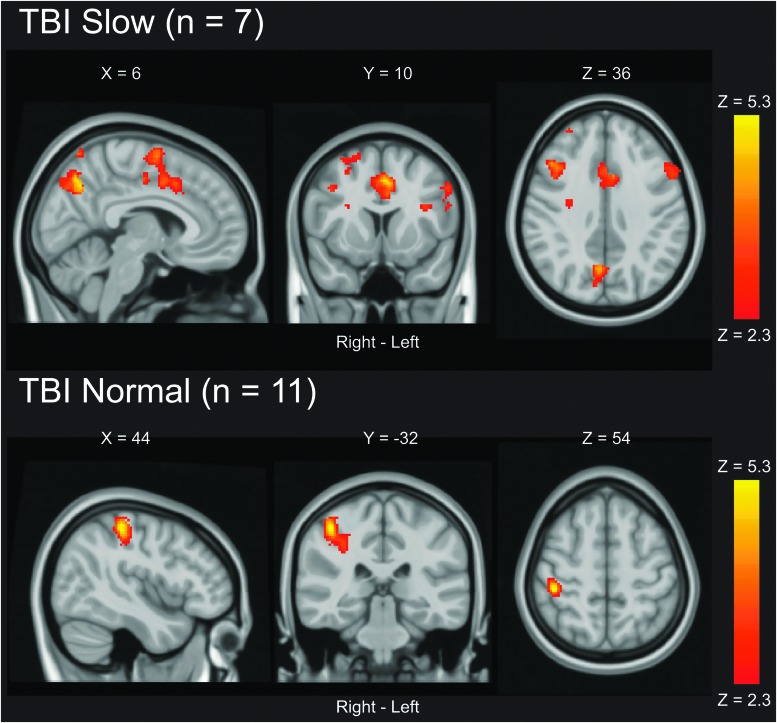
Ad-hoc within-group associations between BOLD activation and the cognitive performance index (CPI). Statistical parametric maps were achieved using a mixed-effects model corrected for multiple comparisons using a cluster threshold of Z > 2.3 and a corrected cluster significance threshold of *p* < 0.05. BOLD, blood oxygen level dependent; TBI, traumatic brain injury.

TBI children with slow and normal IHTT did not differ on the CPI, but both groups performed more poorly than healthy controls. Because the two msTBI groups had similar performance (and did not differ on any other clinical and demographic variables), but were separable based on their levels of activation, we extended our analyses to include ad hoc investigation of within-group associations between BOLD activations and the CPI (Fig. 5). Better cognitive performance as measured by the CPI was associated with pronounced widespread BOLD activation in the slow IHTT msTBI children. In msTBI children with normal IHTT, this relationship was limited to activation in a small region in the post-central gyrus. Positive BOLD-function associations therefore seem stronger within the slow-IHTT children, which provides indirect evidence that hyperactivations observed in the between-group analyses may be compensatory.

The observed BOLD hyperactivations in msTBI children with slow IHTT could not be explained by having sustained a brain injury by itself, nor by injury severity (i.e., GCS). More severe injury as measured by GCS score in the whole TBI sample was associated with BOLD hypoactivations, and TBI patients with normal IHTT exhibited lower WML-dependent BOLD activation compared to healthy controls. TBI children with normal IHTT had statistically significant slower response times at WML 3 and 4 during the fMRI task than healthy controls. Moreover, although not statistically significant, the TBI normal group also showed a trend of slower response times compared to the TBI slow group. This is somewhat counterintuitive, given that one would expect those with slow IHTT to have the slowest response times, especially given that faster IHTT is considered a proxy for better white matter integrity/organization, which may facilitate processing speed.

However, interpretation of differences in response time in the context of similar accuracy on a spatial working memory task is not straightforward. Increased response time also means that the subject must maintain information in their working memory longer (i.e., increased task difficulty) than if they are able to respond faster. Interestingly, earlier research has shown that increased BOLD activation after adult msTBI can be partially attributed to a transient normal response that is also observed in healthy participants attributable to slowed information processing.^6^ BOLD hyperactivations have therefore been suggested to be partly explained by increased on-task “cycle-time,” which is typically reflected in *increased* behavioral response time.^6,7^ If this was the case in our study, the TBI normal group would be expected to show *hyperactivations* rather than *hypoactivations*. It is therefore unlikely that the seemingly compensatory hyperactivations in the slow TBI group are driven by subtle behavioral differences in response time between the groups. However, given that the electrophysiological IHTT measure is a more direct reflection of the basic processing capacity in the brain, BOLD hyperactivation as observed in the TBI slow group may still reflect increased on-task “neuronal cycle time,” possibly representing recruitment of latent support mechanisms that are activated given that the task is more slowly processed on a neuronal level.^6,7^

Our results provide important new directions to interpreting BOLD alterations after pediatric msTBI, but also reflect the highly heterogeneous nature of msTBI, and implies the existence of several potentially non-mutually exclusive mechanisms affecting the BOLD signal (e.g., injury severity, compensatory neuronal processes, or inefficient neuronal processing). Despite including a larger total sample size than most previous fMRI studies of pediatric msTBI, subgroup analysis in our study is still limited by a modest sample size. Our primary aim was to investigate hypotheses regarding the previously identified slow IHTT subgroup, but the statistical power to evaluate more subtle multivariate and possibly non-linear relationships was limited. It is also possible that some of the null findings in our study are type 2 errors, as in similarly powered fMRI studies.^35^

BOLD fMRI studies of pediatric msTBI have now been conducted for just over a decade,^6^ but are still few, and have included sample sizes ranging from 5 to 20 (median, 9) participants.^8^ Larger individual studies are needed, but multi-site collaborative initiatives combining data sets in meta-analyses should also be encouraged to gain critical new knowledge of functional brain adaptations after pediatric msTBI.

In conclusion, children and adolescents with slow IHTT after msTBI exhibit functional brain adaptations expressed as WML-dependent BOLD hyperactivations. Our previous studies in pediatric msTBI have also shown that slow IHTT is associated with widespread white matter disorganization in the post-acute phase,^13^ as well as prolonged neurodegeneration into the chronic phase.^11,12^ The combination of poor white matter organization,^13^ local structural hyperconnectivity,^30^ and slow IHTT^10^ in a subset of children with msTBI may result in the need to recruit a broader network of neurons (i.e., increased BOLD activation) to support cognitive processes that require spatially distributed higher-order, capacity-demanding cognitive activities because of inefficiencies in recruiting more distal processing modules.

Our future analyses will use resting-state fMRI to directly evaluate the impact of poor white matter organization and reduced IHTT on both local and global functional connectivity. Our current results represent a significant step toward a more substantial understanding of the effect of msTBI on the BOLD signal, and the link between hyperactivations and slow IHTT adds to the multi-modal validation of this electrophysiological measure as a promising biomarker.^14^
